# Preparation and Characterization of Lignocellulosic Oil Sorbent by Hydrothermal Treatment of *Populus* Fiber

**DOI:** 10.3390/ma7096733

**Published:** 2014-09-18

**Authors:** Yue Zhang, Sheng Yang, Jian-Quan Wu, Tong-Qi Yuan, Run-Cang Sun

**Affiliations:** 1 Beijing Key Laboratory of Lignocellulosic Chemistry, Beijing Forestry University, Beijing 100083, China; E-Mails: yzhang0504@163.com (Y.Z.); yangsheng113112@126.com (S.Y.); wujq163@126.com (J.-Q.W.); 2 State Key Laboratory of Pulp and Paper Engineering, South China University of Technology, Guangzhou 510640, China

**Keywords:** oil sorbent, hydrothermal treatment, *Populus* fiber, acetylation

## Abstract

This study is aimed at achieving the optimum conditions of hydrothermal treatment and acetylation of *Populus* fiber to improve its oil sorption capacity (OSC) in an oil-water mixture. The characteristics of the hydrolyzed and acetylated fibers were comparatively investigated by FT-IR, CP-MAS ^13^C-NMR, SEM and TGA. The optimum conditions of the hydrothermal treatment and acetylation were obtained at 170 °C for 1 h and 120 °C for 2 h, respectively. The maximum OSC of the hydrolyzed fiber (16.78 g/g) was slightly lower than that of the acetylated fiber (21.57 g/g), but they were both higher than the maximum OSC of the unmodified fiber (3.94 g/g). In addition, acetylation after hydrothermal treatment for the *Populus* fiber was unnecessary as the increment of the maximum OSC was only 3.53 g/g. The hydrolyzed and the acetylated *Populus* fibers both displayed a lumen orifice enabling a high oil entrapment. The thermal stability of the modified fibers was shown to be increased in comparison with that of the raw fiber. The hydrothermal treatment offers a new approach to prepare lignocellulosic oil sorbent.

## 1. Introduction

Oil is one of the most important energy reserves in the contemporary industrial society. It is widely used in petrochemical, transportation, machinery manufacturing, and other important fields [1]. As long as oil is exploited, transported, used and stored, there is a risk of spillage causing significant environmental impact due to tanker disasters, operation failures, equipment damage or natural disasters [2,3,4]. Oil spillage does not only lead to the loss of non-renewable resources, but also threatens the health of marine creatures and human beings. The environmental pollution caused by oil spills has attracted increasing concern, thus, it has become imperative to develop strategies to remedy the oil spillage.

Burning, dispersants, skimmers, oil booms, sorbents and bioremediation are some of the measures that are commonly resorted to solve these oil leakage problems [5]. Wherein, burning and most dispersants can cause secondary pollution, skimmers and oil booms are ineffective for trace oil removal from an oil-water mixture, while bioremediation is efficient but expensive [6,7]. Application of sorbents, however, is an efficient, economical and environmental friendly way to deal with oil spillage [5], not only for the possibility of complete oil cleanup, but also for the convenient post-treatment of oil-loaded sorbents [6]. When it comes to sorbent materials, they usually can be categorized into inorganic mineral materials, synthetic organic materials and organic natural materials [8]. These three kinds of materials have been widely studied for oil removal. Inorganic mineral materials, including clay, perlite, graphite, zeolites, sand and diatomite, show inadequate buoyancy, low oil sorption capacity (OSC) and poor reusability [3,9]. The most often used synthetic organic materials are polypropylene, superhydrophobic sponges and polyurethane [10,11]. These sorbents are quite efficient, but their non-biodegradability is a major disadvantage. Meanwhile, the application of organic natural materials derived from plant sources works as a vital development in sustainable environmental technology [10]. As compared with inorganic mineral and synthetic organic materials, naturally derived sorbents are attractive because of their convenient collection, complete oil removal and simple disposal with minimum environmental hazard [3]. The representative organic natural materials include kapok fiber, wheat straw, sugarcane bagasse, wool fiber, milkweed, rice husks, *etc.* [10,11,12,13,14,15,16,17]. However, most of these raw materials absorb not only oil but also water, which reduces the separation selectivity and efficiency [18]. An ideal sorbent should have highly hydrophobic or lipophilic properties, and as a consequence, a high OSC in an oil-water mixture can be achieved.

Numerous previous works reported that the hydrophobicity of organic natural sorbents could be increased by appropriate modification, such as acetylation, silylation, grafting with oleic acid, thermal carbonization and coating with poly-n-butylmethacrylate (PBMA)/SiO_2_ [4,7,13,19,20,21]. Acetylation with acetic anhydride is one of the simplest, cheapest and safest methods to improve the OSC, strength, dimensional stability and decay resistance of biocomposites among these modifications [22,23]. However, catalysts such as pyridine, dimethylaminopyridine or *N*-bromosuccinimide, are necessary to accelerate the acetylation reaction, which are toxic and expensive for a large-scale application. Taking all these factors into consideration, it is urgent that a new modification approach should be explored for preparing natural sorbents with a high OSC in an economical and environmentally friendly way.

Hydrothermal treatment is an economical and eco-friendly technology since the medium merely contains feedstock and water, and can be applied to all types of lignocellulosic biomass [24]. In the process of hydrothermal treatment, hemicelluloses are hydrolyzed at elevated temperature and pressure, and the hydroxyl groups in the plant cell walls are reduced [25]. Crystalline cellulose is mainly hydrolyzed on its crystal surface, and the internal crystal structure of the cellulose is maintained [26]. As a result, the proportion of crystalline region of the hydrothermal treated material is increased [27,28]. Once the treatment temperature rises above the phase transition temperature of lignin, the lignin droplets deposit on the residual surfaces [29,30]. All these reasons contribute to a decrease of the hydroscopicity of the hydrothermal treated lignocellulosic materials.

In this study, the hydrothermal treatment of *Populus* fiber was applied as a new method to prepare lignocellulosic oil sorbent. The acetylation of *Populus* fiber without any catalysts was also carried out. The optimum conditions of these two different modification methods and the characteristics of the prepared sorbents were comparatively investigated.

## 2. Results and Discussion

### 2.1. Effects of Reaction Conditions on Sorption Capacity

Table 1 shows the OSC of acetylated *Populus* fiber (APF), hydrolyzed *Populus* fiber (HPF) and acetylated *Populus* fiber after hydrothermal treatment (AHPF) under different reaction conditions. It was observed that over 30% of raw *Populus* fiber (RPF) sunk to the bottom of the flask in the oil sorption experiment. RPF had a low OSC (3.94 g/g). Apparently, the acetylation and hydrothermal treatment substantially increased the OSC of *Populus* fiber. The different conditions of both of the treatments were the factors influencing the OSC.

**Table 1:** The effects of different reaction conditions on oil sorption capacity.

Hydrothermal Treatment Condition		Acetylation Condition		Modified Fiber	
Temperature (°C)	Reaction Time (h)	Temperature (°C)	Reaction Time (h)	Sample No.	OSC (g oil/g fiber)
– ^a^	–	–	–	1	3.94
–	–	100	2	2	17.40
–	–	120	2	3	21.57
–	–	140	2	4	19.79
–	–	120	1	5	13.54
–	–	120	3	6	17.74
170	0.5	–	–	7	16.02
170	1	–	–	8	16.78
180	1	–	–	9	16.39
200	1	–	–	10	12.65
220	1	–	–	11	7.66
170	0.5	120	2	12	17.98
170	1	120	2	13	20.30
180	1	120	2	14	19.04
200	1	120	2	15	8.49
220	1	120	2	16	8.33

^a^ Not conducted. Oil sorption capacity (OSC).

The reaction temperature plays a significant role on the effect of acetylation. Upon increasing the acetylation temperature in samples 2–4, the OSC value passed through a maximum, recorded for sample 3, the OSC of 21.57 g/g was 5.5 times that of the unmodified material (3.94 g/g). An increase of temperature from 100 to 120 °C led to an increment of OSC from 17.40 g/g (sample 2) to 21.57 g/g (sample 3). The reason for this change was probably due to the favorable effect of temperature on swellability of the fiber and compatibility of the reagent [31]. Furthermore, extensive hydrogen bonding networks within the matrix formed by the hydroxyl groups of the cell wall polymers were broken during the acetylation process [32]. Increasing temperature favored swelling the fiber, breaking hydrogen bonds, and diffusing the acetylation reagent, thus enhancing the reaction rate and degree, improving the hydrophobic properties of sorbents and then raising the OSC. Of note, a small decrease of OSC was observed when the reaction temperature reached 140 °C, which was in accordance with a previous study [33]. This may be caused by the change of morphology of the fiber.

Table 1 also shows the effect of reaction time on the OSC of APF (samples 3, 5 and 6). The change regularity of the OSC of the fiber with increasing reaction time was similar to the influence of reaction temperature. At 120 °C, an increase of reaction time from 1 to 2 h led to an increment of OSC value by 8.03 g/g, whereas a decrease by 3.83 g/g with duration from 2 to 3 h. This increment of OSC by prolonging the reaction duration was a direct consequence of the favorable effect of time on diffusion and sorption of the reactants between the acetic anhydride and the *Populus* fiber molecules [31]. Acetic acid as the by-product reduced the concentration of acetic anhydride with the extension of time and then decreased the reaction rate [34]. An optimum OSC (21.57 g/g) was assigned to sample 3, prepared by the reaction at 120 °C for 2 h, which served for the following experimental conditions.

The *Populus* fiber was obtained from a fiber board factory with a grinding temperature of 168 °C, so the hydrothermal treatment temperature was started with 170 °C. At 170 °C, the OSC was enhanced at the initial reaction as given by an OSC value of 16.02 g/g for 0.5 h. After that, a slight increase of the OSC of 0.75 g/g occurred when the reaction duration was extended to 1 h. This increment of the OSC value by prolonging the reaction time was the result of the beneficial effect of time on the diffusion of hot-water, dissolution of hemicelluloses and deposition of the lignin droplets. In addition, hydrothermal treatment greatly increased the surface area of cellulose [35]. According to previous studies performed, duration has a trivial influence on hydrothermal treatment efficiency and severity compared with the temperature factor [24]. In order to eliminate the effect of reaction duration for obtaining a higher OSC, 1 h was chosen as the reaction time for the following research.

The reaction temperature has a major effect on the degree of hydrothermal treatment. Insamples 8–11, the OSC value decreased from 16.78 to 7.66 g/g with increasing reaction temperature from 170 to 220 °C. The maximum OSC of HPF (16.78 g/g) was 4.3 times higher than that of RPF (3.94 g/g). In the process of hydrothermal treatment, easily degradable extractives and hemicelluloses are first extracted and separated [36]. Hemicelluloses began to hydrolyze when the temperature was 170 °C followed by lignin dissolution at intermediate temperatures [37]. The lignin droplets deposited on the residual surfaces when the hot water temperature was above the phase transition temperature of lignin [30]. This may be the reason why sample 8 possessed the highest OSC. The amorphous structure of the cellulose decomposed above 180 °C and lignin began to degrade at 190 °C [37]. When the treatment temperature was above 200 °C, the fiber went through a severe swelling progress, and all of the hemicelluloses and much of the lignin dissolved in water at 220 °C to form intermediates [38,39]. Crystalline cellulose hydrolyzed mainly on its crystal surface and therefore the internal crystal structure of the cellulose was maintained intact [26]. The OSC should have been improved due to the increase of hydrophobicity of sorbents, but was reduced instead. This may have been caused by the severe swelling and change of morphology of the fiber. The materials looked like powder rather than fiber. Therefore, the OSC was more strongly influenced by the porous structure of the sorbents rather than by the composition and quantity of dispersed superficial functional groups [40]. In any case, this was a satisfactory result from the economic and environmental point of view since hydrothermal treatment under relatively low temperature would reduce energy cost.

The OSC values of AHPF for samples 12–16 are shown in Table 1. The preparation of AHPF was conducted under the same acetylation conditions after hydrothermal pretreatment of fibers at five different conditions. Therefore, the regularity of the OSC of AHPF was similar to that of HPF, and the development of the trend was also similar. Meanwhile, the OSC values of the pretreatment fiber obtained with and without acetylation were compared. As envisaged, the OSC values of the further acetylated fiber were higher than those without acetylation, except samples 13 to 14. The maximum increment of 3.53 g/g appeared under pretreatment conditions at 170 °C for 1 h. Even so, the general increase of OSC by acetylation was relatively low. When the temperature was below 180 °C, hemicelluloses were removed incompletely and cellulose remained intact. Further acetylation could still convert the acetyl groups to hydroxyl groups, and then improved the hydrophobicity of the sorbents. At 200 °C, cellulose began to decompose, and most of hemicelluloses were dissolved. Instead of raising hydrophobicity, the fiber further degraded under the action of acetic anhydride and by-product acetic acid. Hemicelluloses decomposed to small compounds at the initial lower temperature stage, and then these compounds rearranged through polymerization to form acid-insoluble solid residues above 220 °C [37]. Therefore, it was concluded that it was unnecessary to acetylate the fiber after hydrothermal pretreatment.

### 2.2. FT-IR Spectra

**Figure 1:**
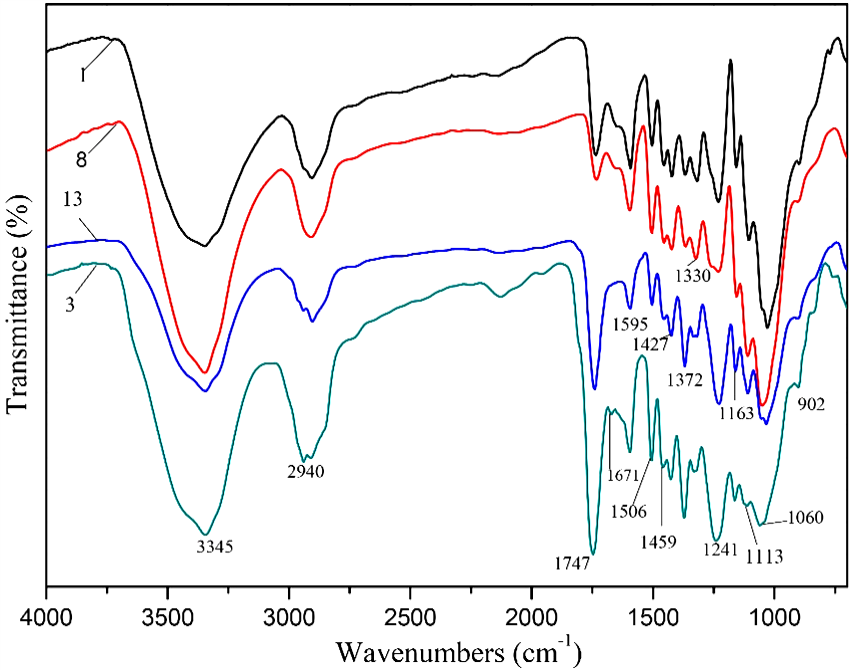
FT-IR spectra of raw *Populus* fiber (RPF) (sample 1), acetylated *Populus* fiber (APF) (sample 3), hydrolyzed *Populus* fiber (HPF) (sample 8), and *Populus* fiber after hydrothermal treatment (AHPF) (sample 13).

FT-IR spectra of RPF (sample 1), HPF (sample 8), AHPF (sample 13), and APF (sample 3) are shown in Figure 1. The detailed assignments of bands in the spectra of untreated and modified *Populus* fiber are listed in Table 2. The acetylated fiber at 120 °C for 2 h (sample 3) is characterized by the occurrence of three important ester bands at 1747, 1372, and 1241 cm^−1^, which are attributed to absorption by carbonyl bonds (C=O in ester), C–H bending vibration (–C–CH_3_), and C–O stretching vibration in esters (–O–C–CH_3_), respectively [6,22]. As compared with sample 1, the absorption intensity of these three ester bands of samples 3 and 13 increased, but those of sample 8 decreased, indicating that ester bonds formed after the acetylation reaction with or without hydrothermal treatment; whereas, in sample 8, most of the acetyl groups were found to be cleaved during the treatment at a high temperature. In samples 3 and 13, the reduced intensity of the band at 1163 cm^−1^ indicated that acetylation cleaved the β-glucosidic linkages but hydrothermal treatment did not. Four intensive bands at 1595, 1506, 1459, and 1427 cm^−1^ correspond to aromatic ring vibrations and ring breathing with C–O stretching in lignin [41]. Obviously, the intensity of these four intensive bands of samples 8 and 13 was reduced, which was attributed to desorption of lignin by washing after hydrothermal treatment. However, sample 3 was similar to sample 1, which indicated that lignin in the wood did not change too much during the acetylation process.

**Table 2:** Assignments of bands in FT-IR spectra of untreated and modified *Populus* fiber.

Band Number	Wavenumber (cm^−1^)	Assignments
1	3345	O–H stretching
2	2940	C–H stretching of –CH_2_– and –CH_3_
3	1747	C=O stretching in ester
4	1595	Aromatic skeletal vibrations plus C=O stretching
5	1506	Aromatic C–C stretching from aromatic ring of lignin
6	1459	Aromatic C–H deformation; asymmetric in –CH_3_ and –CH_2_–
7	1427	Aromatic skeletal vibration combined with C–H in plane deformation
8	1372	C–H bending vibration in –C–CH_3_
9	1330	Phenolic O–H
10	1241	C–O stretching vibration
11	1163	C–O–C vibrations at β-glucosidic linkages in cellulose and hemicelluloses
12	1113	C–O, C–C stretching in cellulose and hemicelluloses
13	1060	C–O stretching in C–O–C linkages
14	902	C–O–C stretching at β-glucosidic linkages

### 2.3. CP-MAS ^13^C-NMR Spectra

**Figure 2:**
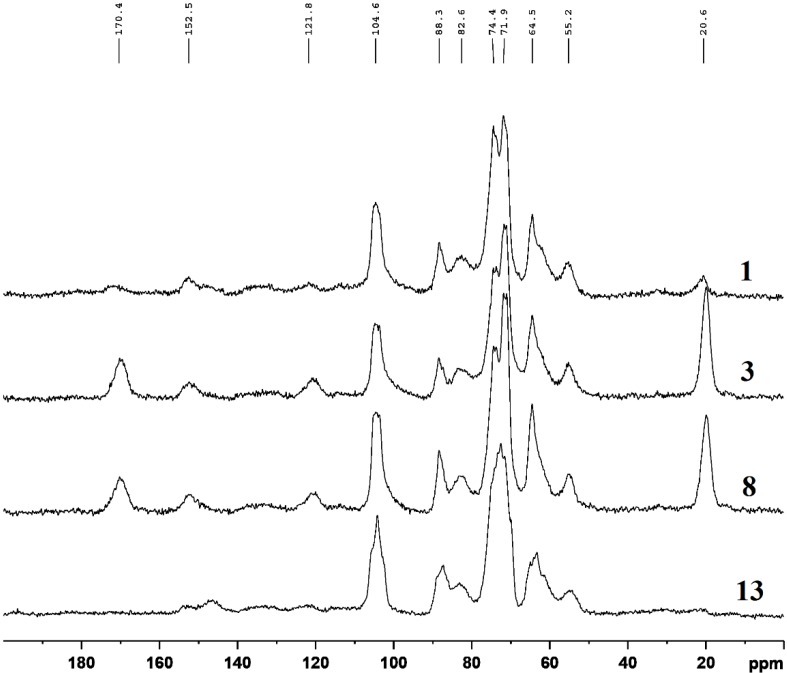
CP-MAS ^13^C-NMR spectra of RPF (sample 1), APF (sample 3), HPF (sample 8), and AHPF (sample 13).

In order to compare the characteristics of the lignocellulosic materials under different treatment conditions, the samples were also subjected to CP-MAS ^13^C-NMR spectroscopy analysis. Figure 2 shows the spectra of raw *Populus* fiber (sample 1) and modified samples 3, 8 and 13. Obviously, the carbohydrate region (55–110 ppm) is dominant and similar in all spectra. The chemical shifts at 88.3 ppm and 64.5 ppm correspond to the crystalline cellulose C-4 and C-6 carbons, respectively [33], while the signal at 82.6 ppm is due to the amorphous cellulose C-4, and a weak signal at 62.9 ppm is attributed to the amorphous cellulose C-6. The sharp signal at 104.6 ppm is assigned to cellulose C-1. The signals at 71–75 ppm are due to cellulose C-2, C-3, and C-5. The signals at 20.6 ppm and 170.4 ppm are attributed to methyl and carboxylic groups of the acetyl functions of hemicelluloses [42]. Evidently, an increase of the relative intensity of both signals at 20.6 and 170.4 ppm in samples 3 and 8 is observed in Figure 2, and a reduction in sample 13 as well.

Corresponding to the FT-IR results, the absorption intensities of three ester bands (carbonyl bonds, C–H stretching and C–O stretching) of samples 3 and 13 increased, while those of sample 8 decreased. It indicated that the acetyl groups of sample 13 observed in Figure 1 formed during acetylation after hydrothermal treatment (sample 13) were converted from the hydroxyl groups of cellulose. Acetyl groups of hemicelluloses cleaved under hydrothermal treatment at 170 °C for 1 h (sample 8). The reason may be that hemicelluloses dissolved and deposited on the fiber surface in the hydrothermal treatment, which were then further washed away by acetone and ethanol after acetylation. The broader region between 110 and 155 ppm is specific for aromatic carbons of lignin [42,43,44,45]. As shown in Figure 2, the relative intensity of the signal at 55.2 ppm, which corresponds to the methoxyl groups of aromatic moieties, was similar under the four different treatment conditions. The signal at 152.5 ppm is assigned to the C-3 and C-5 carbons of S (syringyl) and C-4 carbon of G (guaiacyl) units, and that at 121.8 ppm is assigned to the C-6 carbon of G units [42]. The signals of the samples at 152.5 ppm were almost similar, except sample 13. The intensity of the signal at 121.8 ppm increased in samples 3 and 8. A weak signal at 148.1 ppm, which is attributed to the C-3 and C-5 carbons in non-etherified S units and the C-3 carbons of G units [42], occurred in sample 13. This indicated that S and G units of lignin transformed under the severest conditions.

### 2.4. Morphology Analyses

**Figure 3:**
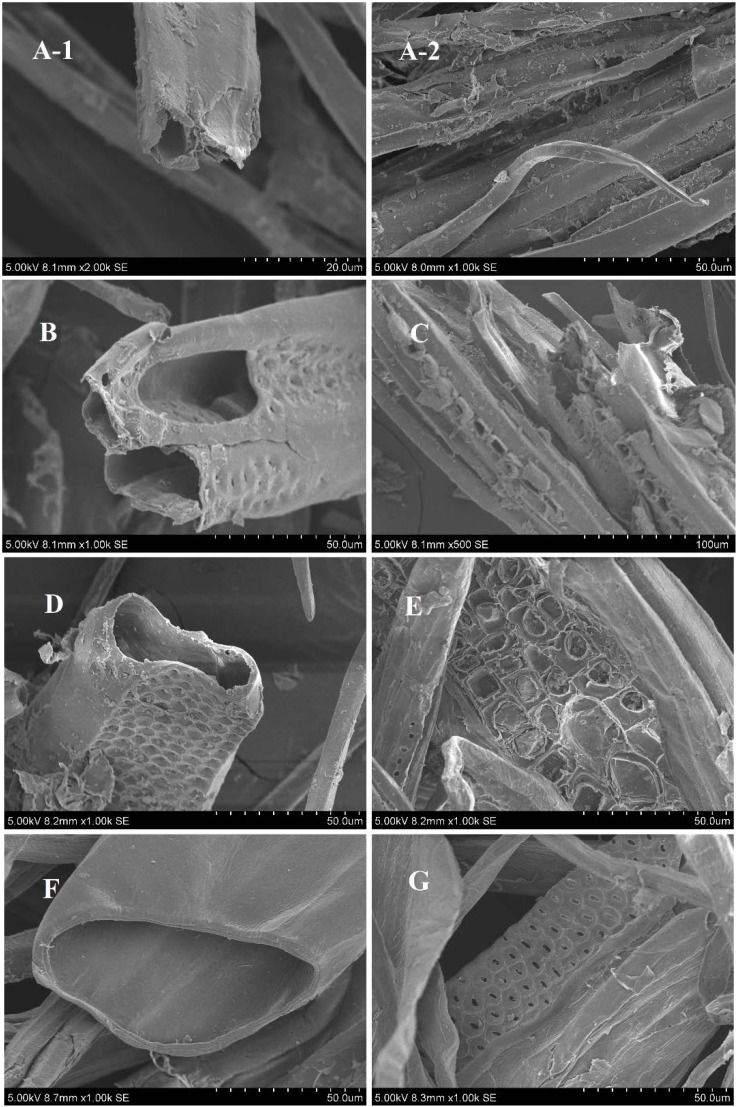
Scanning electron microscopy (SEM) images at various magnifications for untreated and modified *Populus* fiber. (**A**) sample 1; (**B**) sample 3; (**C**) sample 4; (**D**) sample 8; (**E**) sample 9; (**F**) sample 13; and (**G**) sample 14.

The morphology of *Populus* fiber was naturally changed after treatment, which can further affect the oil sorption capacity. Figure 3 shows the surface of *Populus* fiber before and after modification. It is obvious that RPF had a hollow structure and a highly fibrillar morphology with a tiny orifice (Figure 3A-1,A-2 with serious structural breakdown. APF had an intermediate orifice and small amounts of pores. Some cracks were also apparent on the surface, which were ascribed to the swelling function of acetic anhydride and acetic acid (Figure 3B,C). HPF and AHPF showed an open lumen orifice. Numerous micropores and small debris were on the surface, which were attributed to the dissolution of trace amounts of hemicelluloses and lignin (Figure 3D–G). The molten lignin formed at compressed pressure and subsequently condensed. This kind of lumen structure enabled maximum oil entrapment within their empty lumens [3,46]. The rough surface and entangled pore structure of sorbent surfaces provided more active sites for oil adsorption compared to smooth surfaces [47]. The surface of AHPF (Figure 3F) was smoother than HPF (Figure 3D), but the OSC was higher than the latter. The result may be caused by the comprehensive action of the morphology and superficial hydrophobic functional groups of the fiber.

### 2.5. Thermal Analysis

**Figure 4:**
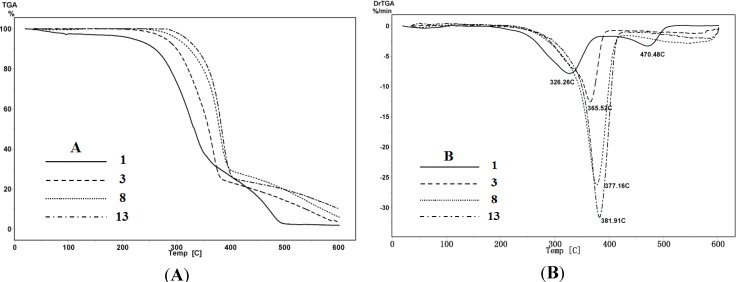
(**A**) Thermogravimetric analysis (TGA) and (**B**) DTG curves of RPF (sample 1), APF (sample 3), HPF (sample 8), and AHPF (sample 13).

The thermal decomposing patterns of original *Populus* fiber and modified samples 3, 8 and 13 were examined by thermogravimetric analysis (TGA) in the temperature range from 25 to 600 °C. TGA and DTG curves of the samples are shown in Figure 4A,B. At 10% weight loss, the decomposition temperature was observed at 218, 278, 300 and 318 °C for sample 1, 3, 8 and 13, respectively. Similarly, at 50% weight loss, the decomposition temperature of RPF and the three modified samples 3, 8 and 13 occurred at 328, 356, 377 and 381 °C, respectively.

Moreover, the maximum DTG in DTG curves (Figure 4B) representing the maximum decomposition rate, could be used to compare the thermal stability of different samples [48]. As illustrated in Figure 4B, DTG_max_ of modified samples 3, 8 and 13 started at 366 °C, 377 °C and 382°C, respectively, while RPF had two DTG_max_ values at 326 °C and 470 °C.

The thermal stability is closely related to the inner structure and the chemical properties of samples. The data indicated that these modified samples were more stable than the raw fiber, and the stability followed the order: sample 3 < sample 8 < sample 13. Among the three major components in wood cell wall, the thermal stability of hemicelluloses was the lowest. Acetylation of the fiber after hydrothermal pretreatment removed hemicelluloses and the amorphous region of the cellulose, leading to an increase of thermal stability.

## 3. Experimental Section

### 3.1. Materials

*Populus* fiber was supplied by a fiber board factory. The *Populus* fiber is principally composed of cellulose (49.0%), hemicelluloses (25.6%) and lignin (23.1%). The fiber was firstly dried in air, and then the powder was removed by a 20-mesh screen while the loose fiber was obtained as raw material. The loose fiber was further dried in a cabinet oven with air circulation for 16 h at 60 °C. The resulting *Populus* fiber was used as raw material (RPF) for further modification and characterization. Acetic anhydride (analytical pure) was obtained from Beijing Chemical Works. Corn oil came from the local commercial market, Beijing, China.

### 3.2. Sample Preparation

#### 3.2.1. Acetylation of *Populus* Fiber (APF)

RPF (2 g) was placed in a round bottom flask equipped with a mechanical stirrer, and then 60 mL acetic anhydride was added. The flask was placed in an oil bath at the required temperature (100, 120 and 140 °C) at atmospheric pressure with a reflux condenser. After the reaction for the required time (1, 2 and 3 h), the flask was removed from the oil bath, and the hot reagent was decanted off. Then the product was filtered and washed with acetone and ethanol to remove unreacted acetic anhydride and acetic acid, and dried in an oven at 60 °C to a constant weight.

#### 3.2.2. Preparation of Hydrothermal Treatment *Populus* Fiber (HPF)

A quantity of RPF (15 g) and 600 mL of distilled water were put into a 1 L stainless steel autoclave. The reactor consisted of a reaction cylinder and a pressure gauge assembly (Parr Instrument Company, Moline, IL, USA). The reactor was heated to the required temperature (170, 180, 200, and 220 °C) and held for 30 or 60 min. Upon completion of the operation, the reactor was cooled to room temperature by the cooling system, then, the device was dismantled and the autoclave was opened. The reaction mixture was filtered to separate the water-soluble and water-insoluble fraction. Then the later was washed with hot water and distilled water until the pH value turned neutral. Then the washed fiber was dried by freeze dehydration for further modification and characterization.

#### 3.2.3. Acetylation of the Fiber after Hydrothermal Pretreatment (AHPF)

The hydrolyzed fiber was further acetylated at 120 °C for 2 h. The specific steps were completed as described in Section 3.2.1.

### 3.3. Characterization of the Modified Fiber

APF, HPF and AHPF were evaluated by FT-IR and solid-state ^13^C-NMR spectroscopies. The FT-IR spectra were collected on a Thermo Scientific Nicolet iN10 FT-IR Microscopy instrument (Thermo Nicolet Corporation, Madison, WI, USA) equipped with a liquid nitrogen-cooled MCT detector. Before data collection, background scanning was performed for correction.

The solid-state CP-MAS ^13^C-NMR experiments were performed at 100 MHz using a Bruker AV-III 400 M spectrometer (Bruker, Karlsruhe, Germany). Each of the dried samples was packed in a 4 mm zirconia (ZrO_2_) rotor, and the measurement was performed using a CP pulse program with 1 ms match time and a 2 s delay between transients. The spinning rate was 5 kHz.

Scanning electron microscopy (SEM) of these samples was carried out with a Hitachi S-3400 N II (Hitachi, Japan) instrument at 15 kV. Before SEM observations, all samples were mounted with conductive glue and coated with a thin layer of gold to improve the conductivity and the quality of the SEM images. The images were obtained at magnifications ranging from 500× to 2000× dependent on the features to be traced.

Thermal stability of the unmodified and modified fiber was performed using thermogravimetric analysis (TGA) on a simultaneous thermal analyzer (DTG-60, Shimadzu, Japan). The sample weighing between 2 and 4 mg in an aluminium oxide crucible was heated from ambient temperature to 600 °C at a heating rate of 10 °C/min. The apparatus was continually flushed with nitrogen.

### 3.4. Measurements of Oil Sorption Capacity

The corn oil (50 g) was mixed with water (250 mL) in a 500 mL conical flask for 10 min at 100 r/min over an orbital shaker. After agitation, the oil floated on the surface of the water and formed an oil layer (with little bubbles). Then, 1.0 g sorbent was added and mixed for 10 min at room temperature. The weight of oil was enough for oil sorption of the fiber. Finally, the sample was removed from the flask using a mesh screen and drained for 3 min. The OSC (Q, g-oil/g-sorbent) of the sorbents was calculated according to Equation (1):

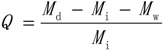
(1)
where, *M*_d_ (g) is the weight of the wet sorbent after draining, *M*_i_ (g) is the initial weight of sorbent and *M*_w_ (g) is the weight of water absorbed in the sorbent. The sorbent after draining was dried in a cabinet oven for 24 h at 105 °C. The weight of sorbent before and after drying was measured, and the difference between the two weights was the weight of water absorbed in the sorbent. All tests were conducted in triplicate and an average value was used.

## 4. Conclusions

In short, the use of hydrothermal treatment instead of acetylation is advantageous because of obtaining an adequate OSC as well as characteristics of economy and environmental compatibility. The optimum condition of the hydrothermal treatment was 170 °C for 1 h, while acetylation was 120 °C for 2 h. The maximum OSC of HPF (16.78 g/g) was 4.3 times that of RPF (3.94 g/g), which was slightly lower than APF (21.57 g/g). Furthermore, acetylation after hydrothermal treatment was unnecessary with the maximum OSC increment of 3.53 g/g. At 10% weight loss, the decomposition temperature of HPF (300 °C) was obviously higher than RPF (218 °C). The thermal stabilities of these modified samples were more stable than the raw fiber. RPF had a hollow structure with a tiny orifice, APF had an intermediate orifice, while HPF and AHPF showed an open lumen orifice. This kind of lumen structure enabled maximum oil entrapment. Consequently, hydrothermal treatment of *Populus* fiber is a promising method for the preparation of oil sorbent.
